# Plasmonic-based impedance microspectroscopy of optically heterogeneous samples

**DOI:** 10.1364/BOE.395474

**Published:** 2020-10-08

**Authors:** Sidahmed A. Abayzeed

**Affiliations:** Optics and Photonics Research Group, Faculty of Engineering, University of Nottingham, Nottingham, NG7 2RD, UK

## Abstract

A robust impedance microscopy technique is presented. This optical tool enables high resolution imaging of electrical properties with promising biophysical applications. The underlying principle is that surface plasmon resonance (SPR) sensors are able to measure perturbations of surface charge density and therefore can be used to compute the impedance of surface-adhered cells. However, the ability to perform reliable quantitative impedance imaging is affected by the optical heterogeneity of the cell-sensor interface. To address this issue, a novel method for quantitative time-resolved resonance angle tracking is developed and applied to correct for the effect of the optical properties. To demonstrate the capability of this technique, impedance microspectroscopy of bovine serum albumin (BSA) patterns was performed enabling measurements of capacitance with submicroscopic resolution. The work presented offers an impedance microspectroscopy method that will create new avenues in studying the electrical properties of single cells and biomolecules as well as bio-electrical currents.

## 1. Introduction

Current biomedical challenges require extensive research efforts to widen the reach of optical microscopy to probe chemical [1,2], mechanical [3] or electrical properties of cells and tissue samples [4]. A significant advancement in sub-microscopic imaging of electrical current was made by Tao and colleagues using surface plasmon resonance (SPR) microscopy [5,6]. Based on this method, a scan-free impedance microscopy technique was developed and applied to study cellular processes such as apoptosis [6] and cell signaling [7]. The method has also been applied, by Gheorghiu’s group, to study molecular interactions in comparison with standard SPR sensing [8]. Impedance spectroscopy of adherent cells, a method that was developed in a pioneering work by Giaever and Keese, demonstrated a great success as a cell assay [9] with applications in monitoring of stem cell differentiation [10], studying signal transduction [11] and characterizing epithelial barrier function [12]. As well as providing complementary information to the present impedance sensing techniques [13,14], the emerging plasmonic-based technique creates new avenues to study electrical heterogeneity at the sub-cellular level.

In a typical impedance spectroscopy system, the impedance of a population of adherent cells is measured using a two-electrode (i.e. working and reference electrode) configuration [14]. A small alternating voltage is applied between these electrodes, for a set of frequencies, while measuring current flow across the sample. The resulting impedance spectrum is used to construct the sample equivalent electrical network [9] and characterise the behavior of the cell population under different conditions. Recently, spatially-resolved impedance spectroscopy has been developed using micro-fabricated electrodes [15] or field-effect transistors [16,17], with a resolution that goes down to a single cell [17]. Extending the capabilities of these approaches to perform measurements at a smaller scale promises enhanced sensitivity and the ability to characterize sub-microscopic structures. Furthermore, performing high resolution impedance measurements using an optical microscopy method would allow the integration with standard microscopy techniques. This, in particular, supports the trend of developing information-rich bioimaging systems with the ability to perform spectroscopic measurements with sub-cellular resolution.

Plasmonic-based optical measurements of the electrical impedance was demonstrated by imaging a pattern of 1-dodecanethiol (DDT) printed on the gold surface [5]. The work was followed by implementing a widefield SPR microscopy method to monitor the dynamic impedance of living cells [6,18]. The underpinning principle of this method is that SPR sensors are sensitive to charge density modulation and therefore are able to detect voltage optically and without fluorescent labels [19,20]. Externally-applied voltage alters the charge density at metal-electrolyte interface which changes the resonance conditions, as a result [19]. Thus, SPR sensors can be seen as two-dimensional electrodes with optical readout in addition to their ability to spatially resolve electrical current.

**Fig. 1.**
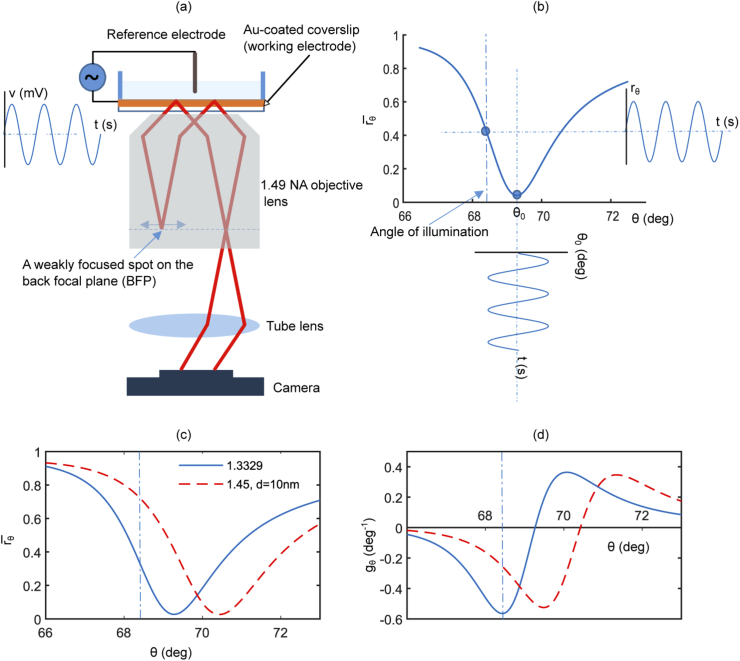
(a) A diagram describes the optical configuration for widefield SPR microscopy. Linearly-polarized light is focused on the BFP to produce a collimated beam on the image plane (i.e. the sensor surface) and the spot on the BFP is translated radially to scan the angle of incidence. A two-electrode configuration is also illustrated where gold thin film is used as an electrode; drawing is not to scale. (b) The sketch shows the transformation of resonance angle modulation to a pixelated change in the reflected intensity, when the sample is illuminated at a fixed angle at the highest gradient of the SPR curve. (c) and (d) show how the response of the sensor (i.e. the gradient $g_{\theta }$ changes with refractive index, for a fixed angle of illumination.)

A widefield SPR microscopy system was used to image local charge density modulation and compute electrochemical impedance at a sub-micrometer scale [6]. In such an optical arrangement [21,22], the sample is illuminated with a collimated beam, fixed at a specific angle of incidence normally at the highest gradient of the SPR curve, as shown in Fig. 1. This approach transforms the changes in resonance angle to pixelated changes in reflectivity in a scan-free manner as illustrated in Fig. 1-(b). Using this setup, a map of electrical impedance of surface-adhered objects can be computed, with the knowledge of the applied voltage (V) and the experimental resonance angle modulation ($\Theta_{0}$) at the angular frequency (ω), using this expression [6]:

(1) $$Z(x,y,\omega )=\frac{V}{j\omega \alpha \Theta_{0}(x,y,\omega )}$$

where α is a parameter that relates the resonance angle modulation to charge perturbation at the sensor-sample interface, j=$\sqrt{-1}$, Z, V and $\Theta_{0}$ are complex quantities. The estimation of the parameter α is discussed in detail in the results section. As described by Eq. (1), one needs to obtain quantitative information on the spatial variation of the voltage-modulated resonance angle. This information is used to compute charge density modulation, given by this term: $\alpha \Theta_{0}(x,y,\omega )$. The measured local charge density provides information about the local impedance of the sample.

Quantifying the time-resolved resonance angle is not straightforward, since the angle scanning methods, which employ minimum finding algorithms [21,22], require recording a series of images and therefore are not suited to quantify the dynamic voltage signals. To overcome this issue, calibration between the resonance position shift and the reflectivity change is determined by Foley et al [5] and validated experimentally. This approach is applicable to specific scenarios where local variations of refractive index are negligible. Since, this calibration depends on the local gradient of the SPR curve, denoted by $g_{\theta }$ in this paper, it changes with the refractive index of the sample as described by Eq. (2). This can be understood by considering the case presented in Fig. 1(c) and (d) where a patterned layer of a dielectric material with a thickness of 10 nm and refractive index of 1.45 is attached to the gold surface in aqueous environment. In this case, the local gradient of the SPR curve (i.e. at a fixed angle of incidence) changes by approximately a factor of 3 when compared to the bare gold in contact with aqueous environment (refractive index 1.3329). This local variation in refractive index results in anomalies in impedance images, if it is not corrected. Accounting for the effect of the refractive index is essential when imaging the electrical properties of optically heterogeneous samples such as cells adhered to gold surface. For instance, inhomogeneous adhesion of cells on the sensor, in addition to variability in the optical properties of the cell membrane, results in a spatially heterogeneous refractive index at the cell-sensor interface. This problem becomes more significant when both optical and electrical properties [6,7] change over time resulting in a time-varying response of the sensor. Therefore, there is still a challenge to develop a resonance angle retrieval method that is suitable to the dynamic measurements of the voltage signal applied in the impedance microscopy. In this brief paper, a new method for quantitative time-resolved measurements of the resonance angle is presented and applied to plasmonic-based impedance imaging.

A novel approach for dynamic plasmonic-based current density microscopy is presented using a gradient mapping technique. The method is described in the following analysis. For the low alternating voltage v(t) that is applied in impedance sensing, the spatio-temporal dynamics of the reflectivity, at a fixed angle of incidence, can be expressed as:

(2) $$r_{\theta }(x,y,t)={\bar{r}}_{\theta }(n)+g_{\theta }(n)h(Z)v(t)$$

where ${\bar{r}}_{\theta }$ is the mean reflectivity at a fixed angle of incidence θ, n=f(x,y) is the spatially-variable refractive index and Z=f(x,y) is the static impedance of the sample. $g_{\theta }$ (1/deg) is the local gradient of the SPR curve, h (deg/V) is a factor that relates the resonance angle modulation $\theta_{0}$ to the applied voltage v(t); it depends on the local impedance Z of the sample. Changes in reflectivity [$\Delta r_{\theta }(n,Z,t)=g_{\theta }(n)h(Z)v(t)$] can be correlated or anti-correlated to the resonance angle change for angles of illumination $\theta <\bar{\theta_{0}}$ and $\theta >\bar{\theta_{0}}$ respectively where $\bar{\theta_{0}}$ represents the mean resonance angle, with the reflectivity change (i.e. gradient) approaches zero at this resonance position, as shown in Fig. 1-(d). Eq. (2) also states that the sensitivity of the method to probe localised electrical properties is affected by the value of the gradient of the SPR curve and therefore by the variations in refractive index, if a fixed angle of illumination is selected (Fig. 1-(d)). In order to obtain the resonance angle modulation, the change in reflectivity is divided by the gradient of the SPR curve at the angle of incidence of choice, as

(3) $$\Theta_{0}(Z)=h(Z)V=\frac{R_{\theta }(n,Z)}{g_{\theta }(n)}$$

Where $R_{\theta }$ is reflectivity modulation obtained by taking the Fourier transform of Eq. (2). For this reason, the gradient of the SPR curve at the angle of illumination (θ) is computed for each pixel to obtain the local response of the sensor and perform localized impedance measurements. Eq. (3) is based on the assumption that gradient does not change over time. This assumption only holds when measuring the static electrical properties of cells and it will be extended to develop a dynamic gradient mapping method in a subsequent work. This method is demonstrated by imaging the impedance of bovine serum albumin (BSA) patterns printed on the gold surface. This sample is selected to test for the effect of refractive index of the sample on the impedance measurements. It is also used to discuss the selection of the angle of incidence, since the variation in the refractive index results in attenuation of the sensitivity of the plasmonic-based impedance method.

## 2. Results and discussions

This section presents the gradient mapping as a method for quantitative time-resolved imaging using SPR microscopy. The method is used to compute the resonance angle modulation while canceling the effect of refractive index. The use of quantitative resonance angle information to calculate localised impedance of the sample is presented followed by discussions on the factors that affect the parameter (α).

### 2.1 Gradient mapping

**Fig. 2.**
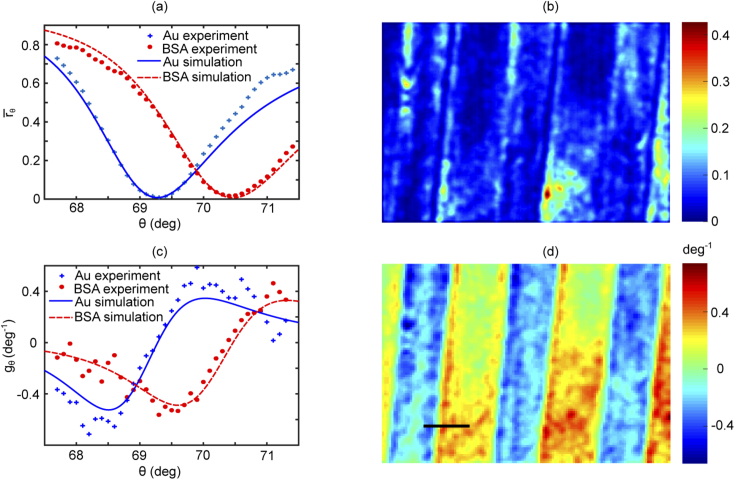
Gradient mapping: (a) SPR curves for bare gold and BSA regions; (b) reflectivity map at θ=69.8°, (i.e. the operating point selected for the impedance measurements); (c) theoretical and experimental gradient of the SPR curves for Au and BSA; and (d) gradient map at θ=69.869.8°. BSA regions can be distinguished from the negative gradient values in (d). Scale bar 8 μm.

Impedance imaging of samples with spatially-variable refractive index, using widefield intensity-based SPR microscopy, requires computation of localised sensor response. This is found by mapping the gradient of the SPR curve at the angle of incidence of choice. This is achieved by stepping the angle of incidence by 0.1° in the range of (±0.3°). The gradient of the SPR curve at this angle is then found from the derivative of the experimental reflectivity as a function of the angle of incidence, as explained in more detail in Appendix C. Results obtained from mapping the gradient of the SPR curve are shown in Fig. 2. For BSA patterns printed on the surface of the gold thin film, SPR curves for both the background and the BSA pattern are presented in Fig. 2(a) fitted to theoretical curves. Theoretical and experimental gradient of the SPR curves are presented in Fig. 2(c), obtained by differentiating the reflectivity with respect to the angle of incidence. Fig. 2(a)-(c) are plotted only for illustration purposes to describe the gradient mapping method and the selection of the angle of incidence, however obtaining these curves is not necessary in an impedance imaging experiment.

Maps of the reflectivity and the gradient are presented in Fig. 2(b) and (d) respectively, for the angle of incidence of ∼69.8° that is used to perform the impedance imaging. These maps show that both the reflectivity and the gradient vary spatially due to the inhomogeneous refractive index of the sample which results in a spatially-variable response to the applied voltage. The contrast in these maps depends on the position of the angle of illumination. It is worth commenting on the selection of the angle of incidence as it is important for sensitive measurements of current density. To perform quantitative measurements of the electrical properties, this angle is set to a point at which both object and background can respond to the applied voltage. This means that the operating point (i.e. the angle of incidence) is not necessarily at the highest gradient for each of the two curves since they are located at two different angles (i.e. 68.568.5°, 69.8° from Fig. 2-(c)) respectively. For instance, if the first operating point (i.e. 68.568.5°) is selected, the system will only report on the electrical properties of the gold electrolyte interface and the response approaches zero at the BSA patterns. For this reason, the operating point is selected at 69.8° where both object and the background can respond to the applied voltage. The approach described above is required, in general, for experiments where signals from single cells are compared to the background.

### 2.2 Quantitative plasmonic-based impedance imaging

**Fig. 3.**
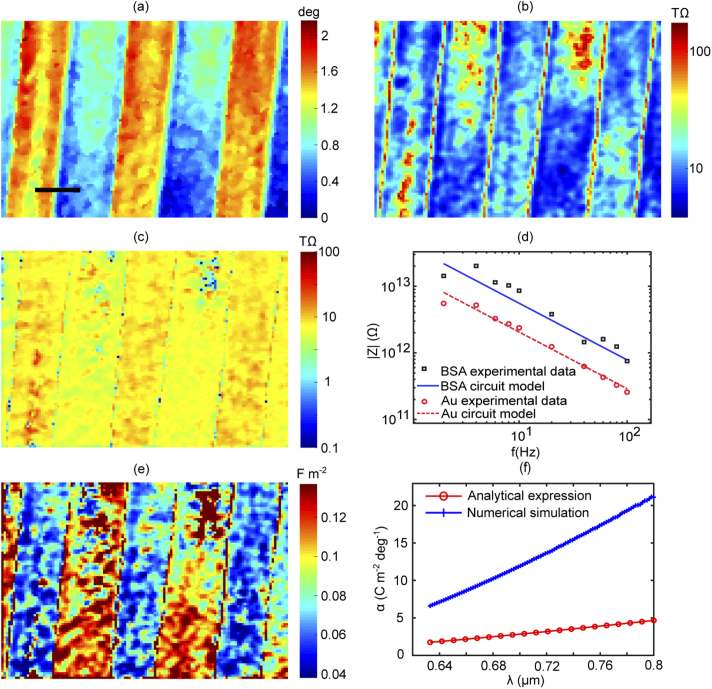
Plasmonic-based imaging of impedance of microscopic structures demonstrated with BSA patterns printed on the sensor surface: (a) quantitative image of the sample showing the static resonance angle that reflects the local refractive index; (b) and (c) are the impedance maps before and after correcting for the effect of the optical properties of the sample, the scale bar is 8μm and the impedance is calculated per pixel - pixel size is 400 nm x 400 nm; (d) microscopic impedance spectrum of BSA and bare gold calculated at a single pixel level; (e) surface capacitance mapped by performing equivalent circuit analysis on impedance microspectroscopy data; and (f) the parameter (α) simulated for a range of wavelengths and compared to the theoretical expression.

Microscopic impedance measurements are degraded by the undesired effects of the local optical properties. In this section, the gradient mapping method is presented as an effective way to suppress the effects of optical properties. A test sample of BSA patterns printed on the gold surface was used to demonstrate this concept. First, a quantitative image of the optical properties of this sample was computed as presented in Fig. 3-(a) by mapping the static resonance angle of the SPR curve. This information is obtained by scanning the angle of incidence and finding the minimum of the SPR curve at each pixel. This procedure gives the average resonance angle, which is not affected by the applied voltage since it only depends on the local optical properties of the sample. In this image, BSA patterns have a higher resonance angle compared to bare gold. Second, Eq. (1) was used to compute the impedance, with the knowledge of the applied voltage and the voltage-modulated resonance angle, as presented in Fig. 3-(b,c). Gradient mapping was implemented using Eq. (3) to obtain quantitative measurements of the voltage-modulated resonance angle. As shown in Appendix D, the latter quantity was used to compute microscopic current flow through the sample.

The effect of the optical properties on the impedance is discussed in Fig. 3-(b,c). The resonance angle modulation that is computed with the assumption of a fixed gradient is presented in Fig. 3-(b) compared to the results of the gradient mapping method shown in Fig. 3-(c). As discussed in the previous section, the response of the sensor to the applied voltage is affected by local optical properties when the sample is illuminated at a fixed angle of incidence. The use of the gradient mapping accounts for the anomalies in the impedance maps due to the effect of the local optical properties. If this is not corrected, the impedance contrast obtained is strongly affected by the local gradient of the sample at the angle of incidence of choice. As observed in Fig. 3-(c) after correcting for the gradient effect, the impedance of the BSA patterns appears higher compared to the background, as one would expect. After this correction, BSA regions showed lower current density, compared to the background (Appendix D). This approach allows both object and the background to be studied simultaneously and can be used to improve the impedance imaging contrast presented in the previous study by Wang et al [6] while enabling a retrieval of reliable quantitative information.

Accurate impedance microspectroscopy offers a novel tool for studying microscopic electrical properties of the sample under the test. In order to investigate the nature of the active and passive components constituting the total impedance, spatially-resolved impedance spectra were produced at the single pixel level. An example of both BSA and gold regions is shown in Fig. 3-(d). These results show that the capacitive properties dominate the impedance of the system in the frequency range from 1-100 Hz. Using the least squares method, both curves were fitted to this expression [23,24]:

(4) $$|Z|=\frac{1}{{\left(j\omega \right)}^{n}Q}$$

where Q is the constant phase element describing the capacitive properties of the interface and n is a factor correcting for the roughness-induced distributed time constant of the interface, it takes values between 0–1 where zero accounts for the case of a pure resistance and 1 describes a pure capacitance. For the sensor used in these experiments, n=0.84 obtained experimentally as described in Appendix D. This analysis is similar to constructing the equivalent circuit of the electrode-electrolyte interface using Randles model. This model represents the electrode as a capacitor in parallel to a charge transfer resistor and the group is in series connection with the solution that is modelled with a resistor. Applying this model to the obtained experimental results, the capacitive route has a dominant effect since it has a lower impedance compared to the charge transfer resistance within the applied frequency range where the contribution of the solution resistance is also negligible.

The data analysis approach described above was performed for each pixel to map the interfacial capacitance presented in Fig. 3-(e). Estimation of double-layer capacitance from constant phase element (Q) is described in Appendix D. Contrast in capacitance between BSA and the gold background is observed. This is expected since the protein deposits have a lower static permittivity compared to water molecules that make the dielectric material of the double layer capacitor of the gold electrolyte interface. The static permittivity of BSA is ∼40 [25] and water has a static permittivity of 80. Another factor that contributes to the observed contrast in the capacitance is the effect of the thickness of the BSA layer (∼10 nm) compared to the size of a water molecule (0.5 nm). A simple estimate of BSA capacitance, assuming the layer forms a parallel-plate capacitor, results in ∼0.04 ${\mathrm{F.m}}^{-2}$ and this value is very close to the BSA capacitance in Fig. 3-(e). The reported capacitance value for gold electrolyte interface is in the range of 0.1 - 0.13 ${\mathrm{F.m}}^{-2}$ close to but lower than the previously reported [5,19]. The presence of contaminants from BSA during the printing process could result in deviation from the anticipated capacitance of the gold-electrolyte interface. These findings are extremely important in the context of biosensing of living cells. For instance, capacitance measurements are performed to characterise cells [10] and study processes such as exocytosis [26]. The ability to perform capacitance imaging promises to provide complementary spatially resolved information on the dynamics of cell membrane.

To convert the voltage-modulated resonance angle to the equivalent charge density map, an analytical expression was derived previously by Foley et al [5] that is given by:

(5) $$\alpha =-\frac{edn_{e}\epsilon_{2}{\left(\epsilon_{1}+\epsilon_{m}\right)}^{2}sin(2\bar{\theta_{0}})}{\epsilon_{1}^{2}(\epsilon_{m}-1)}$$

where e is the unit charge, d is the thickness of the Thomas-Fermi length of the metal film, $n_{e}$ is electron density in the metal film, $\epsilon_{1}$ is permittivity of the glass substrate, $\epsilon_{2}$ is the permittivity of the sample and $\epsilon_{m}$ is the real permittivity of the metal film. Permittivity here refers to the optical permittivity. This expression (Eq. (5) is derived from the commonly-used dispersion relation of surface plasmons of a noble metal with a semi-infinite thickness. However, this dispersion relation does not consider the effect of the thickness of the gold thin film that is commonly used in SPR sensing [27]. The effect of the metal thickness is only included to the expression when the voltage effect on the electron density is considered as explained below. As shown in Eq. (5), the parameter (α) of the sensor depends on the electron density at the metal surface. This effect is confined to the sub-atomic layer of the metal film (i.e. Thomas-Fermi screening length [19]) in contrary to the assumption that voltage affects the electron density of the bulk metal. In this study, the parameter (α) of this sensor is investigated by modeling the voltage effect on SPR sensors combining a multilayer optical model of the sensing structure with the physical chemistry models of the double-layer capacitor at the sensor-electrolyte interface [19]. The results are presented in Fig. 3-(f) compared to the analytical expression Eq. (5) for a series of wavelengths. Other factors such as refractive index of the surface-adhered sample and the applied voltage were investigated but showed a negligible effect. The results presented in Fig. 3-(f) show a strong dependence on the wavelength, the value of (α) obtained from the numerical model for 633 nm is in a good agreement with the experimental value reported by Lu et al (see supplementary material of Ref. [28]). The discrepancy between the numerical modeling and the analytical expression is expected since the analytical expression is based on an approximated dispersion relation of surface plasmons. The value of (α) at 688 nm was used to compute the impedance images presented in Fig. 3.

## 3. Conclusion

The method described here can be applied to perform quantitative imaging of electrical current, either produced by electrically-active cell or used in impedance imaging applications. The reported impedance microscopy method offers a reliable and effective way for mapping electrical properties on a submicroscopic scale while removing the effect of optical heterogeneity. This new capability will have a significant impact on studying bioelectricity as well as dielectric properties of materials. The assumption that gradient does not change with time restricts the applicability of the method to imaging the static electrical properties of single cells. However, to study cellular processes such as proliferation and apoptosis [6] where both electrical and optical properties are time-varying, a dynamic gradient mapping is needed. This approach will be useful to compute the response of the sensor to perform measurements for samples with dynamic refractive index (i.e. time-varying gradient). The temporal correction of the gradient method is also important when studying electrochemical heterogeneity since redox reactions are expected to be associated with refractive index dynamics. The ability to perform localised impedance measurements opens new opportunities to study biological structures and processes that occurs at the subcellular scale as well as molecular interactions.

## Figures and Tables

**Fig. 4.**
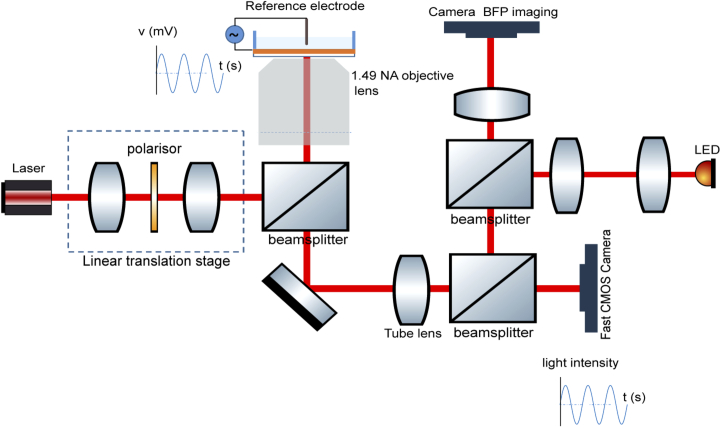
An intensity-based widefield surface plasmon resonance imaging system using a high numerical aperture objective lens.

**Fig. 5.**
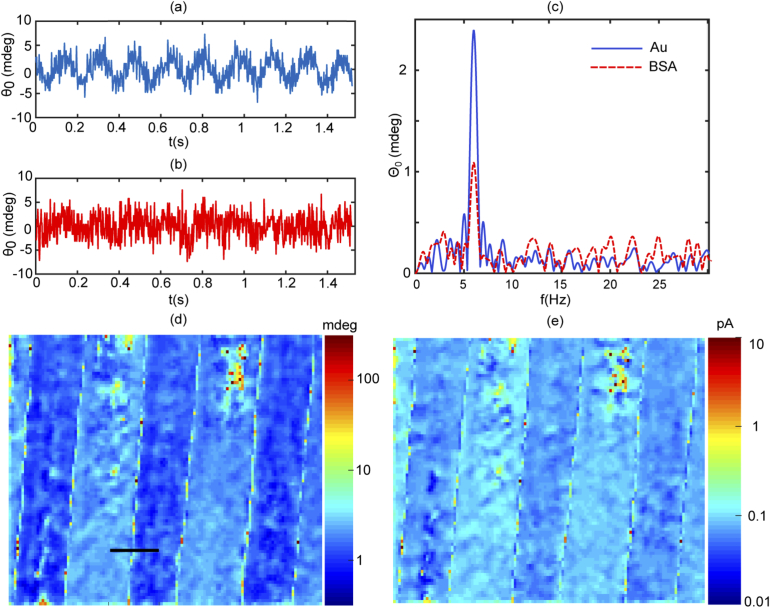
An example for dynamic resonance angle signal is presented in (a) and (b) for Au and BSA respectively. The amplitude of the two signals is shown in (c). Amplitude images of the resonance angle modulation and the current density are shown in (d) and (e) - scale bar 8 μm.

**Fig. 6.**
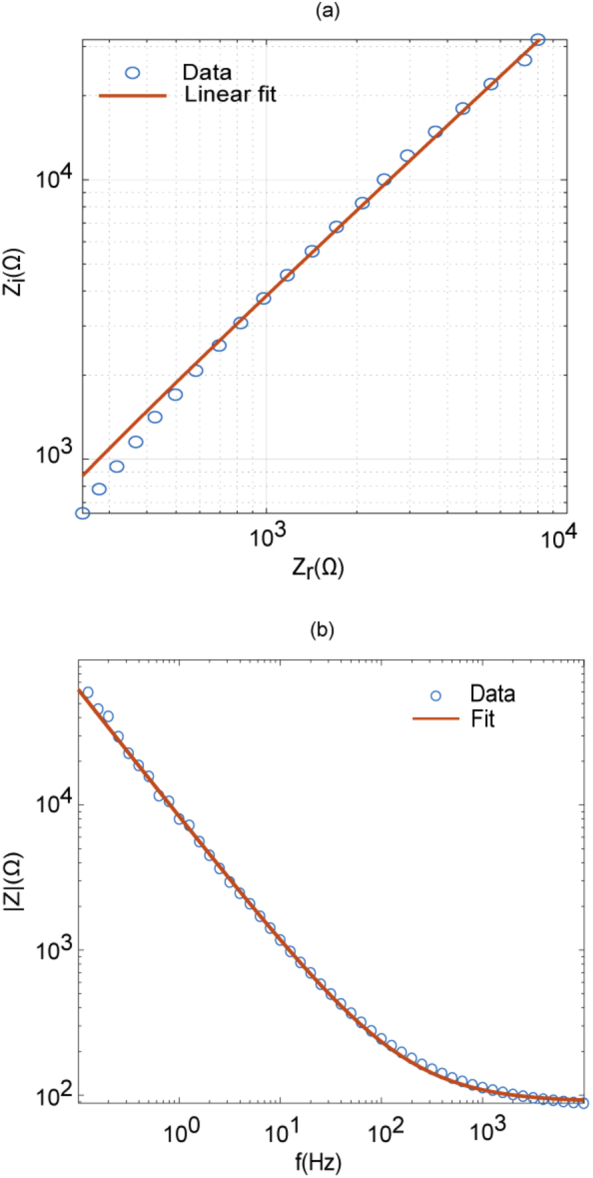
(a) Nyquist and (b) Bode plots for the impedance sensing configuration: SPR sensing film as a working electrode and platinum wire as a reference electrode with 150mM NaCl electrolyte solution.

## Appendix

### A. Time-resolved surface plasmon resonance imaging system

Impedance measurements were performed using the surface plasmon resonance (SPR) microscopy system presented in Fig. 4. The system uses a high numerical aperture objective lens (Nikon NA 1.49 60x). It mainly consists of three optical arrangements: (1) to produce a collimated linearly polarised laser beam to illuminate the sample at a fixed angle of incidence; (2) to image the sample plane on the fast 2D camera (SV643M, EPIX, Inc., IL, US); and (3) using LED source to illuminate the back focal plane (BFP) of the objective lens which is imaged on the 2D camera in Fig. 4. As described, the sample is illuminated with a collimated beam at a specific angle of incidence. To achieve this, a 688 nm fiber-coupled laser beam is collimated before weakly focused on the BFP of the objective lens. This wavelength has been selected so that surface plasmons (SPs) are excited while the SPR curve falls within ranges of angles provided by the numerical aperture of the objective. For this purpose, the BFP of this lens is imaged on a 2D camera to inspect the illumination of the sample. The focused laser beam is translated radially across the BFP of the objective using a linear translation stage to scan the angle of incidence.

### B. Sample preparation and electrode configuration

SPR sensors, used in this study, were prepared by depositing 50 nm of gold on a microscope coverslip (thickness 0.17 mm) using sputtering methods. A 10 nm layer of indium-tin oxide (ITO) was used as an undercoat to promote adhesion of gold to glass without affecting the sensitivity of the SPR sensing structure. A pattern of bovine serum albumin (BSA) was printed on the gold surface using micro-contact printing method. The sample was placed in flow cell that has a platinum reference/counter electrode while the SPR sensor was used as a working electrode. A 150 mM NaCl solution was used as an electrolyte.

### C. Gradient mapping

The gradient of the SPR curve is computed at each pixel, by acquiring a series of images that correspond to small increments of the angle of incidence. This is performed by scanning the focused light on the back focal plane of the objective in steps of 0.1 deg. The reflectivity measurements are fitted to a quadratic function before calculating the gradient. The gradient was found by differentiating the reflectivity (i.e. the reflected intensity normalized to the input intensity) with respect to the angle of incidence.

### D. Signal processing and data analysis

Using a potentiostat (Versastat 3), a sinusoidal voltage waveform (100 mVrms) was applied between the working and the reference electrode while sweeping the frequency in the range of 1-100 Hz. The voltage-modulated reflected light was recorded with a sampling frequency of 300 Hz. The obtained reflected images are normalised to the input intensity before computing fast Fourier transform for the time-resolved reflectivity modulation pixel by pixel, to convert the time domain signal to reflectivity maps for the series of frequencies used in the experiment. The resulting reflectivity data cube is divided by the gradient map to calculate spatially-resolved voltage-modulated resonance angle.

An example of a microscopic real-time signal for an area of 400 nm x 400 nm representing Au and BSA is shown in Fig. 5(a) and (b) respectively. These signals are corrected for the effect of the gradient ($g_{\theta }$) on the reflectivity modulation ($r_{\theta }$) using the following equation:

(6) $$\theta_{0}(t)=\frac{r_{\theta }(t)}{g_{\theta }(n)}$$

These signals are transformed to the frequency domain appearing at 6 Hz in Fig. 5(c), as anticipated. The latter figure forms the basis of the imaging technique where the amplitude of the signal is computed for each pixel to produce quantitative images at a series of frequencies. Amplitude images of dynamic resonance angle and current density at 6 Hz are presented in Fig. 5(d) and (e) respectively. The current density (J) images are calculated by scaling the dynamic resonance angle using:

(7) $$J(x,y,\omega )=j\omega \alpha \Theta_{0}(x,y,\omega )$$

where α is the sensor’s parameter that scales the resonance angle modulation to calculate the underlying charge perturbation at the sensor-sample interface. j=$\sqrt{-1}$, and $\Theta_{0}$ are complex quantities. $\Theta_{0}$ refers to resonance angle modulation and ω is the angular frequency.

Within the frequency range of 1 - 100 Hz, the displacement current through the capacitor of the gold-electrolyte interface is dominant. To map this capacitor, plasmonic impedance microspectroscopy has been performed and the amplitude of the impedance (Z) was fitted to:

(8) $$|Z|=\frac{1}{{\left(j\omega \right)}^{n}Q}$$

where Q is the constant-phase element and ω is the angular frequency.

First, in order to obtain the value of the parameter n, gold-electrolyte interface was studied by performing conventional impedance spectroscopy using a potentiostat (Versastat 3) and the electrode configuration described above. Nyquist plot was produced as presented in Fig. 6(a) and the constant phase angle (ϕ) was found from the slope of the curve with a value of 75.7°. The value of n is therefore 0.84 since it satisfies ϕ/n=90°.

Second, to obtain the double-layer capacitor from the constant phase element Q which dominates the impedance of solid electrode-electrolyte interfaces, a model developed by Brug et al [29] was applied where the double-layer capacitor is found from:

(9) $$C={\left(QR^{\left(1-n\right)}\right)}^{1/n}$$

Where R is the solution resistance. To obtain the value of R, impedance spectroscopy was performed over a range of 0.1 - 10 kHz as presented by the Bode plot in 6(b). The value of R for the experimental configuration described above is 90 Ω was found by fitting the Bode plot to:

(10) $$|Z|=R+\frac{1}{{\left(j\omega \right)}^{n}Q}$$
